# *Toxoplasma gondii* cathepsin proteases are undeveloped prominent vaccine antigens against toxoplasmosis

**DOI:** 10.1186/1471-2334-13-207

**Published:** 2013-05-07

**Authors:** Guanghui Zhao, Aihua Zhou, Gang Lv, Min Meng, Min Sun, Yang Bai, Yali Han, Lin Wang, Huaiyu Zhou, Hua Cong, Qunli Zhao, Xing-Quan Zhu, Shenyi He

**Affiliations:** 1 Department of Parasitology, Shandong University School of Medicine, Jinan, Shandong Province 250012, P R China; 2 Department of Pediatrics, Provincial Hospital Affiliated to Shandong University, Shandong University School of Medicine, Jinan, Shandong Province 250021, P R China; 3 State Key Laboratory of Veterinary Etiological Biology, Key Laboratory of Veterinary Parasitology of Gansu Province, Lanzhou Veterinary Research Institute, CAAS, Lanzhou, Gansu Province, P. R. China

**Keywords:** Toxoplasma gondii, Cathepsin proteases, Bioinformatics, Vaccine, Toxoplasmosis

## Abstract

**Background:**

*Toxoplasma gondii*, an obligate intracellular apicomplexan parasite, infects a wide range of warm-blooded animals including humans. *T. gondii* expresses five members of the C1 family of cysteine proteases, including cathepsin B-like (TgCPB) and cathepsin L-like (TgCPL) proteins. TgCPB is involved in ROP protein maturation and parasite invasion, whereas TgCPL contributes to proteolytic maturation of proTgM2AP and proTgMIC3. TgCPL is also associated with the residual body in the parasitophorous vacuole after cell division has occurred. Both of these proteases are potential therapeutic targets in *T. gondii*. The aim of this study was to investigate TgCPB and TgCPL for their potential as DNA vaccines against *T. gondii.*

**Methods:**

Using bioinformatics approaches, we analyzed TgCPB and TgCPL proteins and identified several linear-B cell epitopes and potential Th-cell epitopes in them. Based on these results, we assembled two single-gene constructs (TgCPB and TgCPL) and a multi-gene construct (pTgCPB/TgCPL) with which to immunize BALB/c mice and test their effectiveness as DNA vaccines.

**Results:**

TgCPB and TgCPL vaccines elicited strong humoral and cellular immune responses in mice, both of which were Th-1 cell mediated. In addition, all of the vaccines protected the mice against infection with virulent *T. gondii* RH tachyzoites, with the multi-gene vaccine (pTgCPB/TgCPL) providing the highest level of protection.

**Conclusions:**

*T. gondii* CPB and CPL proteases are strong candidates for development as novel DNA vaccines.

## Background

The obligate intracellular apicomplexan parasite *Toxoplasma gondii* infects a wide range of warm-blooded animals and causes the anthropozoonotic disease known as toxoplasmosis on a worldwide scale [1,2]. *T. gondii* infection is usually invisible to the host, causing no symptoms or mild ones; however, severe disease complications can occur in immunocompromised individuals and newborns [3,4]. Toxoplasmic encephalitis can emerge as a life-threatening condition in patients infected with the human immunodeficiency virus when reactivation of the cyst stage of *T. gondii* occurs in the brain [5]. In addition, animal studies show that *T. gondii* infection can have a far-reaching influence on host behavior, neuronal function and mate choice. Brain cysts in rats that are chronically infected with *T. gondii* alter the rats’ exploratory and risk taking behavior and unconditioned fear responses, which can lead to greater opportunities for transmission of the parasite from the intermediate rodent host to the definitive host, the cat [6]. *T. gondii* can also enhance the sexual attractiveness of infected male rats [7]. *T. gondii* tachyzoites actively manipulate Ca^2+^ signaling upon glutamate stimulation, leading to neuronal hypo- or hyper-responsivity in the host [8]. Unfortunately, there are no drug treatments available to cure toxoplasmosis. In recent years, the possibility that DNA vaccines, which have the capacity to induce continuous and strong protective immune responses, could be an option for eliminating this ubiquitous parasite has been raised [9,10].

An important design principle for DNA vaccines is the selection of parasite proteins involved in the host cell invasion process by *T. gondii*; therefore, many researchers are working to identify the relevant proteins that may be developed as vaccines against toxoplasmosis [11].

Cysteine proteases play many specialized roles in the body, including endocytosis-related polypeptide degradation [12], tumor invasion [13] and TNF α-induced apoptosis [14]. More importantly, cysteine proteases are important for the growth and survival of apicomplexan parasites that infect humans. *T. gondii* expresses five members of the C1 family of cysteine proteases, including one cathepsin B-like (TgCPB), one cathepsin L-like (TgCPL), and three cathepsin C-like (TgCPC1, 2 and 3) proteases [15]. Among these, TgCPB and TgCPL are mainly expressed in the vacuolar compartment, but a tiny amount of TgCPL has been identified in the late endosome [16-19]. These proteases are thought to function in protein degradation and play specialized roles in the maturation of invasion-related proteins. TgCPB is involved in ROP protein maturation and parasite invasion [16]. In contrast, TgCPL contributes to proteolytic maturation of proTgM2AP and proTgMIC3, and is also associated with the residual body in the parasitophorous vacuole after cell division [20-23].

*T. gondii* cathepsins are considered potential therapeutic targets based on the results of the following genetic and inhibitor studies: antisense inhibition of TgCPB expression or treatment with cathepsin inhibitors diminished parasite replication, cell invasion and infection *in vivo*[16,24]; genetic disruption of TgCPL diminished parasite cell invasion and growth [17]; and, the cathepsin inhibitor morpholinurea-leucyl-homophenyl-vinyl sulfone phenyl inhibited parasite invasion by blocking the release of invasion proteins from microneme secretory organelles [25,26]. To our knowledge, no studies have described the induction of protective immune responses against *T. gondii* CPB and CPL in the host*.* We propose that a DNA vaccine construct based on TgCPB and TgCPL could be a useful tool against disease caused by *T. gondii*.

Hence, in this study, we used bioinformatics approaches to analyze TgCPB and TgCPL, the results of which identified a large number of linear-B cell epitopes and potential Th-cell epitopes on these proteases. This suggested the possibility that TgCPB and TgCPL could be used as vaccines. Based on the results of the epitope analyses, pTgCPB and pTgCPL were constructed as single-gene vaccines and pTgCPB/TgCPL as a multi-gene vaccine, and their immunogenicity, protective efficacy, and potential as vaccine candidates against *T. gondii* infection were examined in laboratory mice.

## Methods

### Prediction of protein secondary structure and linear-B cell epitopes

Epitopes are the foundation of protein antigenicity that determines antigen specificity [27,28]. There are many types of epitope prediction methods in use, including hydrophilicity, accessibility, antigenicity, flexibility, charge distribution and secondary structure [29-34]. Despite the lack of an infallible method to predict antigenic epitopes, several rules can be followed to determine which peptide fragments of a protein are likely to be antigenic. Firstly, antigenic epitopes should be located in solvent-accessible regions and contain both hydrophobic and hydrophilic residues. Secondly, peptides lying in long loops connecting secondary structure motifs should be selected preferably, while peptides located in helical regions should be avoided. Whenever possible, peptides that are in the N- and C-terminal regions of the protein should be chosen because they are usually solvent accessible and unstructured.

According to the rules outlined above, we analyzed the linear-B cell epitopes of TgCPB and TgCPL using DNAStar software and chose peptides that have good hydrophilicity, high accessibility, satisfactory flexibility and strong antigenicity. Thereafter, we used DNAMAN software to search for linear-B cell epitopes in the TgCPB and TgCPL amino acid sequences.

### Prediction of Th-cell epitopes

*T. gondii* is an obligate intracellular parasite; hence, cellular immunity mediated by T cells plays an important role in *T. gondii* infection [35]. To develop an effective vaccine against toxoplasmosis, it is necessary to elucidate which type of Th cell-mediated immune response is necessary. Predicting Th cell epitopes is currently rather complicated and the results are ambiguous; however, there are some rules that can be used to predict Th cell epitopes [36,37]. Here, we used the Immune Epitope Database (http://tools.immuneepitope.org/analyze/html/mhc_II_binding.html) online service to predict the half maximal inhibitory concentration (IC_50_) values of peptides binding to the major histocompatibility complex (MHC) class II molecules of TgCPB and TgCPL. We also used SYFPEITHI (http://www.syfpeithi.de/Scripts/MHCServer.dll/EpitopePrediction.htm) to determine the ligation strength to a defined HLA (or H2) type for TgCPB and TgCPL. Note that such binding to MHC is necessary but not sufficient for recognition by T cells.

### Parasites and mice

Female 6-week-old BALB/c mice were purchased from Shandong University Laboratory Animal Center. All mice were maintained under specific pathogen-free conditions. All of the animal experiments were approved by the Ethics Committee on Animal Experiments of the Medical School of Shandong University.

The *T. gondii* RH strain was harvested from the peritoneal fluid of the BALB/c mice 72 h after infection, and was washed by centrifugation and resuspended in sterile PBS. Half of the *T. gondii* tachyzoite suspension was used to extract total RNA and genomic DNA, while the other half was used to prepare soluble tachyzoite antigens (STAg) using an ultrasonic disintegrator. STAg preparations were aliquoted and stored at −80°C until use.

### Construction of expression plasmids

The whole TgCPB gene was amplified from *T. gondii* total RNA by reverse transcription polymerase chain reaction (PCR) using the two primer pairs shown below. TgCPB for prokaryotic expression used the following primers plasmid construction: 5′-cgGAATTCATGGAGGGGCGAAAGTC-3′ (forward) and 5′-ccgCTCGAGCTACATTTCTCTCTCCTCTTCTG-3′ (reverse), both of which contain *Eco*RI/*Xho*I restriction sites (underlined). Plasmid construction for eukaryotic expression of TgCPB consisted of 5′-ataagaat GCGGCCGCATGGAGGGGCGAAA-GTC-3′ (forward) and 5′-ccgCTCGAGCTACATTTCTCTCTCCTCTTCTG-3′ (reverse), both of which contain *Not*I/*Xho*I restriction sites (underlined).

The whole TgCPL gene was PCR amplified from of *T. gondii* genomic DNA using the two primer pairs described below. Prokaryotic expression plasmid construction for TgCPL used the following primers: 5′-cgGAATTCATGGACAGCAGCGAGACGC-A-3′ (forward) and 5′-ccgCTCGAGTCACATCACGGGGAAAGACG-3′ (reverse); *Eco*RI and *Xho*I restriction sites are underlined. Eukaryotic expression plasmid construction for TgCPL used the following primers: 5′-ccAAGCTTATGGACAGCA-GCGAGACGCA-3′ (forward) and 5′-gcTCTAGATCACATCACGGGGAAAGACG-3′ (reverse); *Hin*dIII and *Xba*I restriction sites are underlined.

The PCR products for both genes were cloned into a pEASY-T1 vector (TransGen Biotech, China) to generate a recombinant cloning plasmid. After sequencing, TgCPB and TgCPL were subcloned into a eukaryotic expression plasmid pET-30a(+) (Novagen, USA) and the prokaryotic expression plasmid, pBudCE4.1 (Invitrogen, USA) to produce pET30a-TgCPB, pET30a-TgCPL, pBudCE4.1-TgCPB and pBudCE4.1-TgCPL. Finally, TgCPB and TgCPL were concurrently subcloned into the prokaryotic expression plasmid pBudCE4.1 to produce pBudCE4.1-TgCPB-TgCPL.

### TgCPB and TgCPL expression in *Escherichia coli* and antigen production

pET30a-TgCPB and pET30a-TgCPL constructs were used to transform *E. coli* BL21(DE3) cells, which were then grown in Luria-Bertani medium with kanamycin (25 μg/mL). Synthesis of recombinant TgCPB and TgCPL proteins was induced using 1mM isopropyl-β-D-thiogalactoside for 6 or 8 h at 25°C. The cells were then lysed and centrifuged at 4°C for 15 min at 10,000 × *g*. Recombinant proteins were then purified via binding of their carboxy terminal histidine tags to Ni-NTA resin (Sangon Biotech, China).

Experimental mice were subcutaneously immunized with 100 μg of purified rTgCPB or rTgCPL prepared in equal volumes of Freund’s complete adjuvant for the first immunization. The second and third immunizations contained 50 μg of the purified protein in Freund’s incomplete adjuvant. Samples of antisera were collected 2 weeks after the last immunization.

### Examination of antibody specificity by western blotting

Sodium dodecyl sulfate-polyacrylamide gel electrophoresis (SDS-PAGE) and western blotting were used to investigate antibody specificity, as described previously [38]. STAg preparations were removed from the ultra-low temperature freezer and 500 ng of each preparation was used for SDS-PAGE. The separated protein bands were transferred onto polyvinylidene difluoride (PVDF) membranes (Millipore, USA), each of which was blocked with 5% w/v skimmed milk powder diluted in PBS for 2 h at room temperature before separate incubation with mouse anti-TgCPB or anti-TgCPL antibodies, or pre-immune mouse sera (dilution 1:600). After a wash in PBS-Tween 20, each of the membranes was incubated with diluted goat anti-mouse IgG horseradish peroxidase (HRP)-labeled secondary antibody (1:10,000; Sigma, USA) for 1h. Parasite proteins were visualized using electrochemiluminescence reagents (Cowin Biotech, China).

### TgCPB and TgCPL expression in mammalian cells

When the cell density reached 80–90%, HEK293 cells were transfected with pBudCE4.1-TgCPB or pBudCE4.1-TgCPL using Lipofectamine™ 2000 reagent (Invitrogen, USA). After 24-h incubation, the cells were fixed with cold methanol for 20 min and protein expression was evaluated using an indirect fluorescence antibody test as previously described [39]. Briefly, anti-TgCPB or anti-TgCPL antibodies were used as primary antibodies and a FITC-labeled goat anti-mouse IgG antibody (ZSGB-Bio, China) was used as the secondary antibody. After rinsing three times with PBS, the coverslips were immediately observed under a fluorescence microscope (Carl Zeiss, Germany). The cells were then lysed with RIPA Lysis Buffer (50 mM Tris, pH 7.4; 150 mM NaCl; 1% Triton X-100; 1% sodium deoxycholate; 0.1% SDS) containing 1 mM of the protease inhibitor phenylmethanesulfonyl fluoride, after which they were centrifuged at 12,000 × *g* for 10 min, at either 24 h or 48 h post-transfection.

### SDS-PAGE and western blot analysis

Protein production from HEK293 cells was monitored by SDS-PAGE and western blotting. About 500 ng of the purified rTgCPB or rTgCPL proteins were separated using SDS-PAGE. The separated protein bands were transferred onto PVDF membranes. The detailed procedures are the same as in the above section “Identification of the antibody specificity by western blotting”.

### Animal experiments

Five groups of BALB/c mice (n = 13 each) were individually injected 4 times at two-weekly intervals with one of the following: PBS, pBudCE4.1, pTgCPB, pBudCE4.1-TgCPL or pBudCE4.1-TgCPB-TgCPL. Two weeks after the final immunization, the mice were challenged by intraperitoneal (i.p.) injection of 100 μL of PBS containing 1 × 10^4^*T. gondii* tachyzoites and the survival time of each mouse was recorded.

### Antibody assays

The levels of IgG antibodies against *T. gondii* were analyzed using an enzyme-linked immunosorbent assay (ELISA) [40]. The microtiter plates (Costar, USA) were coated with STAg (10 pmol/well) and incubated at 4°C overnight. After washing three times with PBS-T, the plates were blocked with 1% bovine serum albumin for 1 h at 37°C. The plates were washed a further three times and incubated with the mouse sera diluted in PBS for 1 h at 37°C. After three washes, secondary goat anti-mouse IgG, IgG1 or IgG2a conjugated with HRP (Sigma) was added and incubated at 37°C for 1 h. Immune complexes were revealed by incubating with ortho-phenylenediamine (Sigma) and 0.15% H_2_O_2_ for 30 min. Reactions were stopped by the addition of 2 M H_2_SO_4_ and read at 490 nm with an ELISA plate reader (EL800; Bio-Tek, USA). All samples were run in triplicate.

### Cytokine assays

Cytokine levels were detected according to the previously described method [39]. Briefly, three mice per group on week 4 after the final immunization were euthanized and their spleens removed under sterile conditions. Viable splenocytes were dispensed into 96-well plates at 37°C in 5% CO_2_ and the cell-free supernatants were harvested and assayed for IL-4 levels at 24 h, or at 72h for IL-10, or at 96 h for IFN-γ using an ELISA kit (R&D Systems, USA).

### Statistical analyses

Statistical analyses were performed using SPSS software. Antibody production and cytokine levels among the different groups were determined using a one-way analysis of variance. Survival times in the mice were compared using the Kaplan-Meier method. Values of P < 0.05 were considered statistically significant.

### Ethics statement

All experimental procedures using animals in the present study had received prior approval by the Institutional Animal Care and Use Committee of Shandong University under Contract 2011–0015. Humane endpoints to reduce pain or distress in the experimental animals were employed via euthanasia. Mice were monitored daily over 8 weeks for signs of toxoplasmosis, which included difficulties with their food and water intake, lethargy, or severe ascites. Mice that showed signs of illness were sacrificed immediately using CO2 gas; this involved placing the mice in a chamber and administering CO2 at a concentration of 60% to 70% over a 5-minute exposure time, after which the cervical dislocation method was sometimes used to ensure that effective euthanasia had occurred.

## Results

### Prediction of linear-B cell epitopes and Th-cell epitopes

The results of the prediction analyses indicated the presence of 21 potential epitopes on TgCPB and 17 on TgCPL, as shown in Tables 1 and 2. The Th-cell epitopes on TgCPB and TgCPL that were identified by bioinformatic analyses are predicted to have the ability to bind strongly to MHC class II molecules (Tables 3 and 4). The binding strength of the interaction is known to influence the direction of Th cell differentiation, where, as the binding force increases, more cells tend to differentiate into Th-1 cells [41,42]. As such, we speculate that TgCPB and TgCPL are likely to induce Th-1 cell-mediated immune responses.

**Table 1:** Linear-B cell antigenic epitope prediction for TgCPB

**Order**	**Amino acid position**	**Potential antigenic sequences**	**Score**
1	325–346	LMPLSAQHTTSCCNAIHCASFG	1.190
2	6–30	SFRVLGTPLPFAALAAILLLGCMYT	1.183
3	57–86	AEDVLNAFVSPESVESLFDSIVAEQVVATS	1.176
4	379–407	CWPYEVPFCAHHAKAPFPDCDATLVPRKT	1.175
5	118–127	GELLRLLLAD	1.170
6	147–160	RHIVRDSVLVSEKA	1.164
7	283–294	AFPACKDVVGHV	1.157
8	453–489	GPVSGAFMVYEDFLSYKSGVYKHVSGLPVGGHAIKII	1.153
9	196–204	SNAAVALIK	1.130
10	300–306	CGSCWAF	1.108
11	243–250	GTFLVNTK	1.103
12	225–236	EVSLRFRYLSLK	1.092
13	272–278	EPVPAHF	1.091
14	314–320	DRLCIRS	1.084
15	38–44	DSLFPLS	1.078
16	434–440	TSAYSLR	1.067
17	139–145	FRHLTHS	1.061
18	420–426	ADNVHPF	1.060
19	362–367	KGVVTG	1.055
20	260–265	MPLPAK	1.045
21	520–525	MGQCGI	1.039

**Table 2:** Linear-B cell antigenic epitope prediction for TgCPL

**Order**	**Amino acid position**	**Potential antigenic sequences**	**Score**
1	60–82	RAWIALVAAAVSLLVFASFLIQW	1.205
2	364–372	DHGVLLVGY	1.168
3	142–151	KNNLVYIHTH	1.152
4	303–320	RAQSCEKVVKILGFKDVP	1.152
5	89–100	AVFPPSPVEDHQ	1.152
6	35–44	PSPPFVVTTR	1.152
7	187–209	KSHHLGVATELLNVLPSELPAGV	1.141
8	410–419	QCGLLLDASF	1.138
9	214–221	RGCVTPVK	1.136
10	6–12	THYVSFL	1.130
11	328–341	KAALAKSPVSIAIE	1.120
12	278–297	FQYVLDSGGICSEDAYPYLA	1.119
13	239–255	EGAHCAKTGKLVSLSEQ	1.110
14	226–232	CGSCWAF	1.108
15	347–362	FQFYHEGVFDASCGTD	1.100
16	113–127	FQDAFSSFQAMYAKS	1.050
17	175–180	RKYLGF	1.035

**Table 3:** **IC**_**50 **_**values for TgCPB and TgCPL peptide binding to MHC class II molecules obtained using the immune epitope database**

**MHC II Allele**^**1**^	**Start-Stop**^**2**^	**Sequence**^**3**^	**Percentile Rank**^**4**^
TgCPB			
HLA-DRB1*01:01	10–24	LGTPLPFAALAAILL	0.01
HLA-DRB1*01:01	71–85	ESLFDSIVAEQVVAT	6.21
HLA-DRB1*01:01	228–242	LRFRYLSLKDAKKLM	4.99
HLA-DRB1*01:01	320–334	SQGKRLMPLSAQHTT	2.74
HLA-DRB1*01:01	470–484	SGVYKHVSGLPVGGH	1.58
H2-IAb	8–22	RVLGTPLPFAALAAI	3.83
H2-IAb	59–73	DVLNAFVSPESVESL	3.51
H2-IAb	187–201	VFWESRPASSNAAVA	0.91
H2-IAb	300–314	CGSCWAFASTEAFND	4.46
H2-IAb	549–563	LPGQRAAGARAGAHA	3.19
H2-IAd	15–29	PFAALAAILLLGCMY	1.88
H2-IAd	191–205	SRPASSNAAVALIKK	4.34
H2-IAd	227–241	SLRFRYLSLKDAKKL	1.21
H2-IAd	320–334	SQGKRLMPLSAQHTT	3.19
H2-IAd	428–442	QDTHKATSAYSLRSR	2.52
H2-IEd	182–196	ETGEDVFWESRPASS	17.89
H2-IEd	226–240	VSLRFRYLSLKDAKK	13.43
H2-IEd	351–365	QPGMAWRWFERKGVV	4.77
H2-IEd	399–413	DATLVPRKTPKCRKD	16.82
H2-IEd	432–446	KATSAYSLRSRDDVK	9.89
TgCPL			
HLA-DRB1*01:01	60–74	RAWIALVAAAVSLLV	0.04
HLA-DRB1*01:01	46–60	YFWKKFLRQRNFTAR	6.00
HLA-DRB1*01:01	117–131	FSSFQAMYAKSYATE	2.74
HLA-DRB1*01:01	136–150	RRYAIFKNNLVYIHT	3.24
HLA-DRB1*01:01	178–192	LGFKKSRNLKSHHLG	5.20
H2-IAb	27–41	RRGVRAGRPSPPFVV	1.49
H2-IAb	59–73	ARAWIALVAAAVSLL	3.37
H2-IAb	85–99	EDDRAVFPPSPVEDH	3.42
H2-IAb	116–130	AFSSFQAMYAKSYAT	5.68
H2-IAb	226–240	CGSCWAFSTTGALEG	2.49
H2-IAd	22–36	GELHQRRGVRAGRPS	6.17
H2-IAd	57–71	FTARAWIALVAAAVS	0.14
H2-IAd	117–131	FSSFQAMYAKSYATE	2.63
H2-IAd	318–332	DVPRRSEAAMKAALA	0.01
H2-IAd	395–409	RDGYMYMAMHKGEEG	1.82
H2-IEd	42–56	TTRTYFWKKFLRQRN	7.05
H2-IEd	174–188	RRKYLGFKKSRNLKS	13.03
H2-IEd	202–216	PSELPAGVDWRSRGC	28.28
H2-IEd	309–323	KVVKILGFKDVPRRS	21.70
H2-IEd	376–390	KESKKDFWIMKNSWG	24.70

^1^ H2-IAb, H2-IAd and H2-IEd alleles are mouse MHC class II molecules; the HLA-DRB1*01:01 allele is a human MHC class II molecule.

^2^ We chose 15 amino acids for analysis each time.

^3^ TgCPB or TgCPL sequences.

^4^ Low percentile = high binding.

**Table 4:** Ligation strength analysis of TgCPB and TgCPL for MHC class II molecules using SYFPEITHI

**MHC II Allele**^**1**^	**Start-Stop**^**2**^	**Sequence**^**3**^	**Score**^**4**^
TgCPB			
H2-Ad	55–69	FSAEDVLNAFVSPES	31
H2-Ad	76–90	SIVAEQVVATSGNLT	27
H2-Ad	412–426	KDCEEQAYADNVHPF	27
H2-Ad	117–131	AGELLRLLLADSEDM	26
H2-Ak	43–57	LSEDTSVDPRESFSA	24
H2-Ak	72–86	SLFDSIVAEQVVATS	24
H2-Ak	149–163	IVRDSVLVSEKAFPS	24
H2-Ak	37–51	DDSLFPLSEDTSVDP	22
H2-Ed	313–327	NDRLCIRSQGKRLMP	28
H2-Ed	436–450	AYSLRSRDDVKRDMM	28
H2-Ed	229–243	RFRYLSLKDAKKLMG	26
H2-Ed	309–313	TEAFNDRLCIRSQGK	24
H2-Ek	86–100	SGNLTESAPRDRDSA	24
H2-Ek	313–327	NDRLCIRSQGKRLMP	22
H2-Ek	463–477	EDFLSYKSGVYKHVS	22
H2-Ek	37–51	DDSLFPLSEDTSVDP	20
HLA-DRB1*0101	4–18	RKSFRVLGTPLPFAA	35
HLA-DRB1*0101	13–27	PLPFAALAAILLLGC	34
HLA-DRB1*0101	470–484	SGVYKHVSGLPVGGH	33
HLA-DRB1*0101	71–85	ESLFDSIVAEQVVA T	32
TgCPL			
H2-Ad	60–74	RAWIALVAAAVSLLV	31
H2-Ad	68–82	AAVSLLVFASFLIQW	30
H2-Ad	192–206	GVATELLNVLPSELP	30
H2-Ad	58–72	TARAWIALVAAAVSL	27
H2-Ak	18–32	GLENGELHQRRGVRA	22
H2-Ak	146–160	VYIHTHNQQGYSYSL	22
H2-Ak	196–210	ELLNVLPSELPAGVD	22
H2-Ak	256–270	ELMDCSRAEGNQSCS	20
H2-Ed	311–325	VKILGFKDVPRRSEA	30
H2-Ed	44–58	RTYFWKKFLRQRNFT	26
H2-Ed	170–184	RDEFRRKYLGFKKSR	26
H2-Ed	369–383	LVGYGTDKESKKDFW	26
H2-Ek	310–324	VVKILGFKDVPRRSE	24
H2-Ek	77–91	SFLIQWQGEDDRAVF	22
H2-Ek	365–379	HGVLLVGYGTDKESK	22
H2-Ek	6–20	THYVSFLNGEDDGLE	20
HLA-DRB1*0101	59–73	ARAWIALVAAAVSLL	34
HLA-DRB1*0101	60–74	RAWIALVAAAVSLLV	31
HLA-DRB1*0101	195–209	TELLNVLPSELPAGV	31
HLA-DRB1*0101	308–322	EKVVKILGFKDVPRR	30

^1^ H2-Ad, H2-Ak, H2-Ed and H2-Ek alleles are mouse MHC class II molecules; the HLA-DRB1*0101 allele is a human MHC class II molecule.

^2^ We chose 15 amino acids for analysis each time.

^3^ TgCPB or TgCPL sequences.

^4^ High score = high binding.

### Prokaryotic and eukaryotic expression vectors for TgCPB and TgCPL

**Figure 1:**
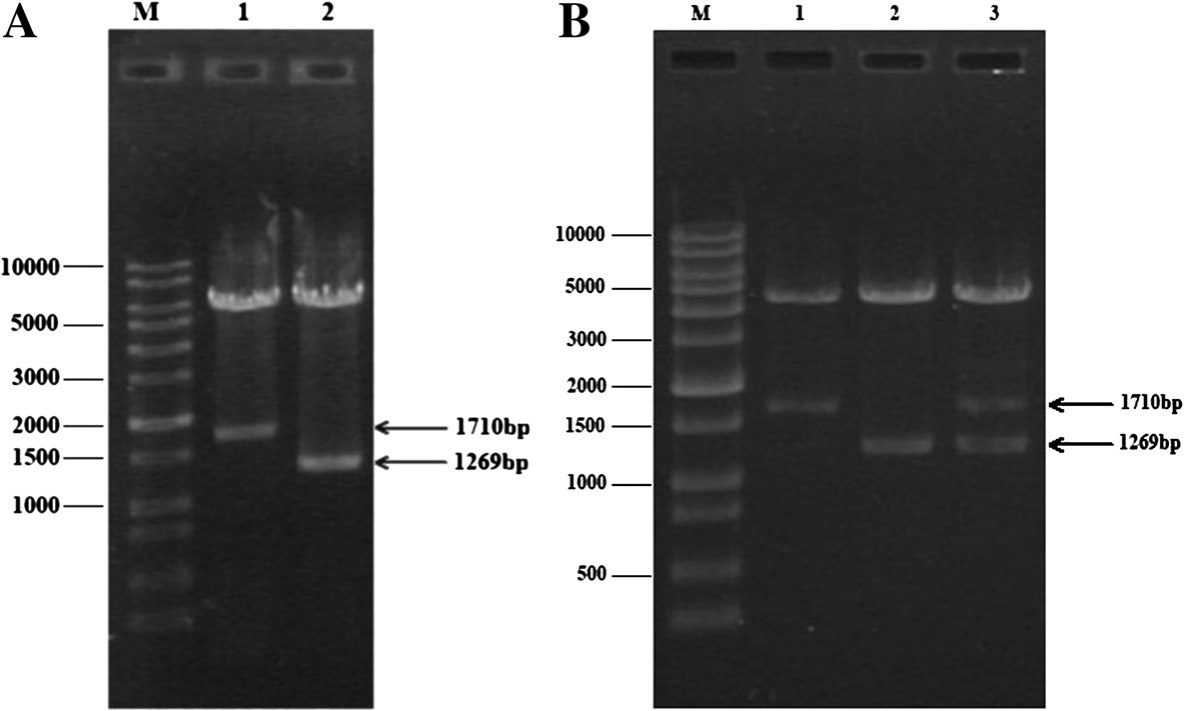
**Recombinant cathepsin protease expression plasmids.** (**A**) M: DNA size marker; Lane 1: recombinant pET30a-TgCPB plasmid digested with *Eco*RI and *Xho*I; Lane 2: recombinant pET30a-TgCPL plasmid digested with *Eco*RI and *Xho*I. (**B**) M: DNA size marker; Lane 1: recombinant pBudCE4.1-TgCPB plasmid digested with *Not*I and *Xho*I; lane 2: recombinant pBudCE4.1-TgCPL plasmid digested with *Hin*dIII and *Xba*I; lane 3: recombinant pBudCE4.1-TgCPB-TgCPL plasmid digested with *Not*I, *Xho*I, *Hin*dIII and *Xba*I.

Recombinant plasmids pET30a-TgCPB and pET30a-TgCPL were digested with *Eco*RI and *Xho*I resulting in the correct sized fragments and indicating successful construction of the prokaryotic expression plasmids (Figure 1A). Similarly, restriction digests of pBudCE4.1-TgCPB (*Not*I and *Xho*I), pBudCE4.1-TgCPL (*Hin*dIII and *Xba*I), and the dual construct pBudCE4.1-TgCPB-TgCPL (*Not*I, *Xho*I, *Hind*III and *Xba*I) confirmed that the recombinant eukaryotic expression plasmids had been correctly made (Figure 1B).

### Antibody specificity against rTgCPB and rTgCPL proteases

**Figure 2:**
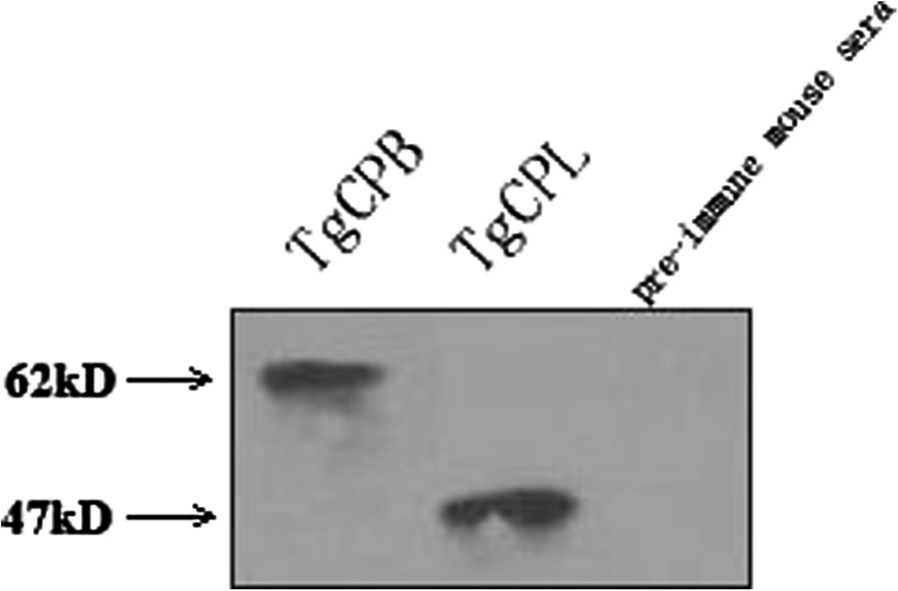
Western blot identification of TgCPB and TgCPL proteins in tachyzoite-stage parasites.

PVDF membranes from western blots of STAg preparations were incubated separately with mouse anti-TgCPB or anti-TgCPL antibodies or pre-immune mouse sera. The results showed that the mouse anti-TgCPB and anti-TgCPL antibodies recognized proteins bands of about 62 kDa and 47 kDa, which is consistent with the expected sizes of the TgCPB and TgCPL proteins, respectively (Figure 2).

### Identification of protein expression in HEK293 cells using immunofluorescence assay (IFA) and western blotting

**Figure 3:**
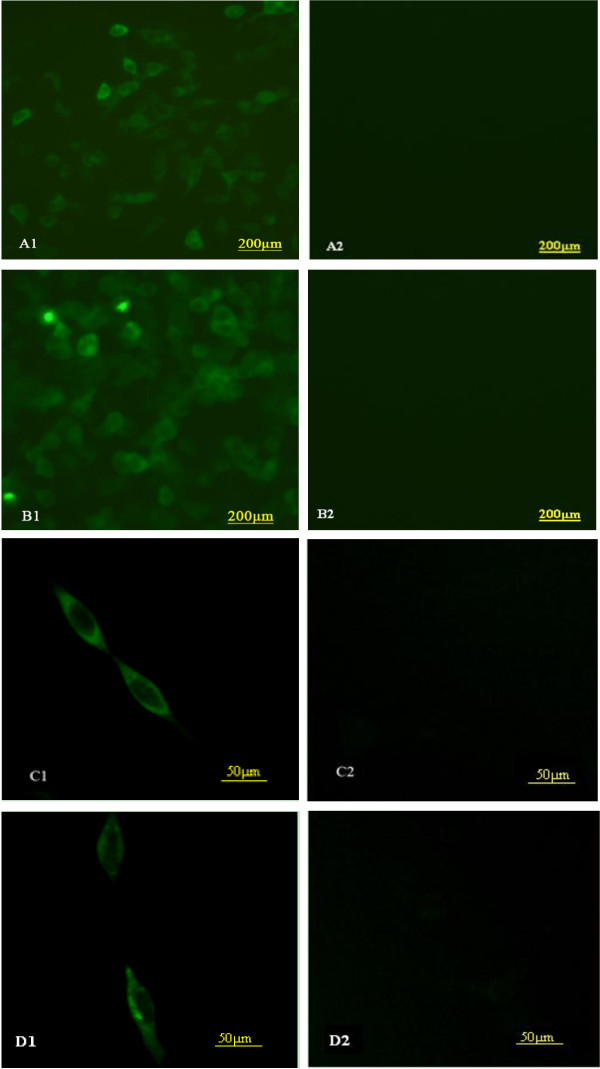
**Indirect fluorescent antibody detection of recombinant TgCPB and TgCPL proteases on the surface of HEK293 cells.** (**A1**) pTgCPB-transfected HEK293 cells; (**A2**) pBudCE4.1-transfected HEK293 cells. (**B1**) pTgCPL-transfected HEK293 cells; (**B2**) pBudCE4.1-transfected HEK293 cells. (**C1**) pTgCPB/TgCPL-transfected HEK293 cells where pTgCPB/TgCPL expression was detected using the anti-TgCPB antibody as the primary antibody; (**C2**) pBudCE4.1-transfected HEK293 cells where the anti-TgCPB antibody was used as the primary antibody. (**D1**) pTgCPB/TgCPL-transfected HEK293 cells where the anti-TgCPL antibody was used as the primary antibody; (**D2**) pBudCE4.1-transfected HEK293 cells where the anti-TgCPL antibody was used as the primary antibody. High level of laser intensity was used for A1, A2, B1 and B2, lower level of laser intensity for C1, C2, D1 and D2.

**Figure 4:**
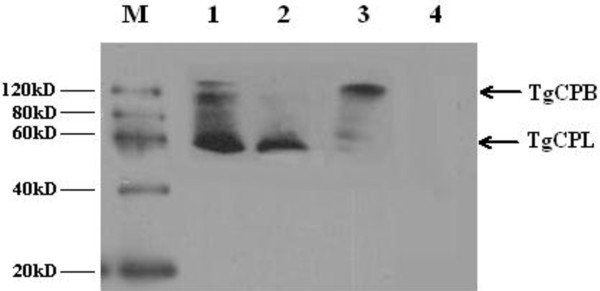
**Western blot analysis of TgCPB and TgCPL protein expression in transfected HEK293 cells.** M: protein marker; (1) HEK293 cells transfected with the recombinant pTgCPB/TgCPL plasmid; (2) HEK293 cells transfected with the recombinant pTgCPL plasmid; (3) HEK293 cells transfected with the recombinant pTgCPB plasmid; (4) HEK293 cells transfected with an empty vector.

*In vitro* expression of pTgCPB, pTgCPL and pTgCPB/TgCPL were evaluated by IFAs at 48h post-transfection. As shown in Figure 3, green fluorescence was observed in HEK293 cells, whereas no fluorescence was observed in the pBudCE4.1 vector transfected cells. Western blotting analysis confirmed expression of rTgCPB (~62kDa) and rTgCPL (~47kDa) in HEK293 cells transfected with pTgCPB or pTgCPL. Both proteins were detected in cells transfected with the dual expression vector, pTgCPB/TgCPL (Figure 4).

### Antibody responses in immunized mice

**Figure 5:**
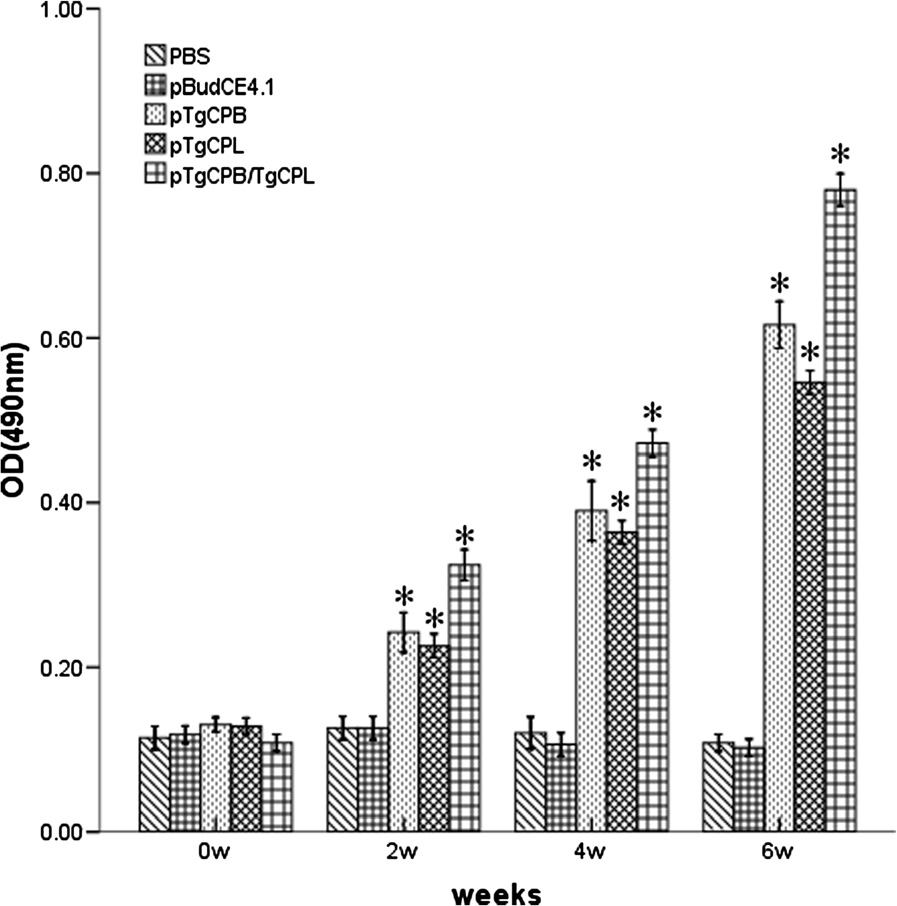
**Determination of specific IgG antibodies in sera of immunized mice.** Sera from 13 mice per group were collected one day prior to each immunization and the subtype levels were determined using enzyme-linked immunosorbent assays. All samples were run in triplicate. The results are representative of 3 experiments and shown as the mean of the OD_490_ ± SD. *, statistically significant differences (P < 0.05) compared to the phosphate-buffered saline (PBS) or pBudCE4.1 controls.

High levels of immunoglobulin G (IgG) antibodies were observed in the experimental mice immunized with pTgCPB, pTgCPL, or pTgCPB/TgCPL. The antibody levels gradually increased with successive immunizations and were higher than those of the control groups, which were immunized with phosphate-buffered saline (PBS) or pBudCE4.1 (Figure 5). A significant difference was detected between the experimental groups and the control groups (P < 0.05). IgG antibody levels in the pTgCPB/TgCPL group were higher than those of the pTgCPB and pTgCPL groups (P < 0.05). However, no statistical difference was detected between the pTgCPB and pTgCPL groups (P > 0.05). These results indicate that the recombinant plasmids encoding TgCPB and TgCPL induced strong IgG antibody responses in the mice. Furthermore, the OD value for this group reached a high level two weeks after the third immunization.

**Figure 6:**
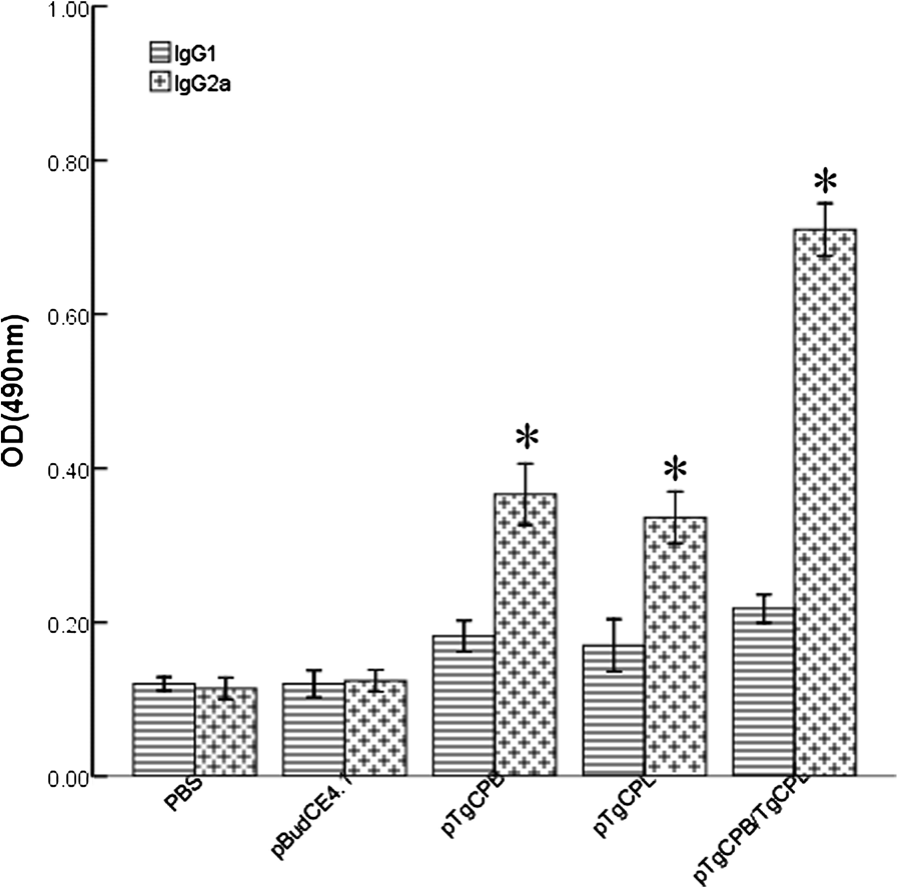
**Distribution of IgG subtypes IgG1 and IgG2a in immunized mice.** Sera from 13 mice per group were collected from 2^th^ week after the final immunization and the subtype levels were determined using enzyme-linked immunosorbent assays. All samples were run in triplicate. IgG subtypes IgG1 and IgG2a levels in the sera of mice two weeks after the last immunization were analyzed using an enzyme-linked immunosorbent assays. The results are representative of 3 experiments and expressed as the mean of the OD_490_ ± SD. *, statistically significant differences (P < 0.05) compared to the control groups.

IgG subclass (IgG1 and IgG2a) levels in all of the groups during the second week after the final immunizations were investigated to determine whether a Th1- or Th2-type response was elicited (Figure 6). An apparent predominance of IgG2a over IgG1 was observed in both the single-gene or multi-gene vaccine immunized mice, indicating a shift toward a Th1-type response. Furthermore, mice immunized with pTgCPB/TgCPL generated higher IgG2a levels than those immunized with pTgCPB or pTgCPL alone (P < 0.05). However, there was no significant difference in the IgG2a levels between the pTgCPB and pTgCPL groups (P > 0.05).

### Cytokine production

The splenocyte supernatant was harvested at different times and used to measure cytokine production (interferon-γ [IFN-γ], interleukin-4 [IL-4] and IL-10) in the different groups. As shown in Table 5, mice vaccinated with pTgCPB/TgCPL generated significantly higher levels of IFN-γ than mice vaccinated with pTgCPB, pTgCPL, PBS, or an empty vector (P < 0.05). The level of IFN-γ in the pTgCPB-immunized mice was higher than that of the pTgCPL-immunized mice, but the difference was not statistically significant (P > 0.05). In addition, the low levels of IL-4 and IL-10 seen in the experimental and control groups suggested no statistically significant differences among the groups (P > 0.05). IFN-γ and IL-2 favor Th1-type immune responses, whereas IL-4 and IL-10 favor Th2-type responses. These results show that the cellular immune response induced by the single- or multi-gene vaccines tended to be a Th1-type response in the mice.

**Table 5:** **Cytokine production by splenocyte**^**a **^**cultures from immunized BALB/c mice**

**Group**	**Cytokine production (pg/mL)**^**b**^		
	**IFN-γ**	**IL-4**	**IL-10**
PBS	47.59 ± 4.63	37.26 ± 2.84	44.34 ± 2.77
PBudCE4.1	48.35 ± 1.86	33.70 ± 3.29	38.70 ± 2.70
pTgCPB	674.93 ± 83.36^*^	32.58 ± 3.72	34.60 ± 1.92
pTgCPL	585.14 ± 112.03^*^	32.34 ± 3.87	35.47 ± 1.94
pTgCPB/TgCPL	1182.23 ± 94.28^*#^	36.11 ± 3.51	34.57 ± 2.14

IFN, interferon; IL, interleukin, PBS, phosphate-buffered saline.

^a^ Splenocytes were obtained from 3 mice per group and were collected at week 4 after the final immunization.

^b^ Values for IFN-γ at 96 h. Values for IL-10 at 72 h. Values for IL-4 at 24 h.

*Compared with the PBS-adjuvant or pBudCE4.1 controls (P < 0.05); # compared with pTgCPB or pTgCPB (P < 0.05).

### Protective efficacy of DNA vaccination against *T. gondii* in mice

**Figure 7:**
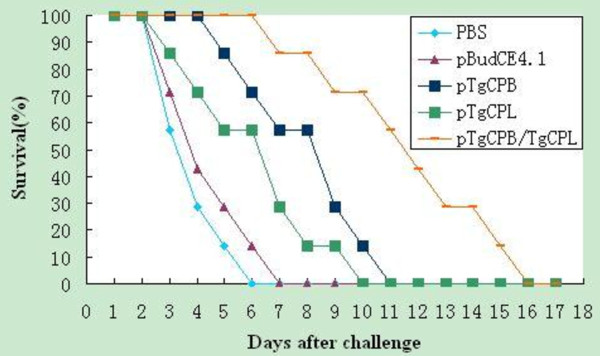
**Survival curves of the vaccinated BALB/c following *Toxoplasma gondii *challenge infections.** 10 mice per group were challenged with 1 × 10^4^ tachyzoites of the virulent *T. gondii* RH strain on the 4^th^ week after the final immunization. Survival was monitored daily for 18 days post-challenge with the parasites.

To evaluate the level of immunoprotection induced by the DNA vaccines, all of the mice were challenged intraperitoneally with the *T. gondii* RH strain and mortality was monitored daily until all of the mice showed signs of illness and were killed (Figure 7). Mice immunized with the DNA vaccines had dramatically higher survival times than did the control groups vaccinated with PBS or pBudCE4.1 (P < 0.05). Mice vaccinated with pTgCPB/TgCPL showed a greater survival time than those vaccinated with pTgCPB or pTgCPL (P < 0.05). However, no significant difference was observed between mice immunized with pTgCPB and those immunized with pTgCPL (P > 0.05).

## Discussion

Bioinformatics is an established interdisciplinary science related to mathematics, statistics, computer science, physics, biology and medicine [43]. Because of its effectiveness and low cost, it has been widely used to predict the structure, function and antigenic epitopes of proteins by estimation of the similarity of the protein of interest to a sequence of known structure or function [44,45]. As previously described, we initially used DNAStar software to analyze the secondary structures of TgCPB and TgCPL, followed by DNAMAN software to analyze their sequences from which we identified many good liner-B cell epitopes. Secondly, we used two online services (IEDB and SYFPEITHI) for analyzing Th-cell epitopes and found several potential Th-cell epitopes on TgCPB and TgCPL.

Through the animal experiments, we confirmed that both TgCPB and TgCPL can induce strong humoral and cellular immune responses and noted a significantly higher level of total IgG antibodies, IgG2a, and IFN-γ than that observed for the controls. These results show that TgCPB and TgCPL make good vaccine antigens, thus highlighting the reliability of the bioinformatics approaches that were used herein.

In the present study, we successfully constructed pTgCPB and pTgCPL single-gene vaccines and a pTgCPB/TgCPL multi-gene vaccine. Both single- and multi-gene vaccines produced humoral and cellular immune responses in the murine host. The multi-gene vaccine was superior to the single-gene vaccine; it elicited stronger immune responses and more effective protection against *T. gondii* infection. Importantly, all of the mice in the experimental groups immunized with pTgCPB, pTgCPL, or pTgCPB/TgCPL displayed strong humoral immune responses as shown by their high IgG levels. The high levels of IgG2a and IFN-γ and low levels of IL-4 and IL-10 suggest that the cellular immune responses were mediated by Th-1 cells [46,47]. These experiments were conducted in BALB/c mice; however, it would be interesting to test other strains of mice with different MCH backgrounds to investigate the range of immune responses to the vaccines.

The survival times of all of the mice in the five groups after intraperitoneal challenge with 1 × 10^4^ tachyzoites of the virulent RH strain of *T. gondii* were recorded. Compared to the mice in the control groups, the immunized mice showed protection against *T. gondii* infection: all mice in the control groups showed signs of illness and were killed within 8 days post-challenge, whereas mice immunized with pTgCPB, pTgCPL, or pTgCPB/TgCPL had significantly increased survival rates. Mice immunized with pTgCPB/TgCPL showed stronger humoral and cellular immune responses and significantly prolonged survival times than mice in the pTgCPB and pTgCPL groups. All of the mice showed signs of illness and were killed by day 16 post-challenge. The results indicate, therefore, that the DNA vaccines did not provide complete protection against *T. gondii* RH tachyzoite infection.

Finally, the pBudCE4.1 vector should be mentioned. This vector was chosen to for expression of the multi-gene vaccine because it has two promoters (CMV and EF-1α), thus ensuring that TgCPB and TgCPL can both be expressed whilst avoiding mutual interference.

## Conclusions

In this study, we used bioinformatics approaches to identify antigenic epitopes on TgCPB and TgCPL proteases. Thereafter, we made single-gene (pTgCPB and pTgCPL) and multi-gene (pTgCPB/TgCPL) DNA vaccines to evaluate the level of immunoprotection induced in mice immunized with such vaccines. The experimental results are consistent with the bioinformatics predictions that the antigenic epitopes on these proteins should induce appropriate immune responses. Hence, these results show that bioinformatics analyses to predict antigenic epitopes on proteins can be a useful tool for vaccine research. When the vaccinated mice were given a challenge infection with *T. gondii* RH tachyzoites, we found that the DNA vaccine constructs did not provide complete protection against infection, however. Nevertheless, DNA vaccines merit further investigated as a strategy for controlling *T. gondii* infection.

## Competing interests

The authors declare that they have no competing interests.

## Authors’ contributions

GZ carried out the experiments and drafted the manuscript. GL and MM revised the manuscript. YB, MS, YH, LW, QZ, HZ, HC and XQZ conducted the experiments and revised the manuscript. AZ and SH conceived and designed the study and revised the manuscript. All of the authors have read and approved the final manuscript.

## Pre-publication history

The pre-publication history for this paper can be accessed here:

http://www.biomedcentral.com/1471-2334/13/207/prepub
